# Actin Cytoskeleton as Actor in Upstream and Downstream of Calcium Signaling in Plant Cells

**DOI:** 10.3390/ijms20061403

**Published:** 2019-03-20

**Authors:** Dong Qian, Yun Xiang

**Affiliations:** MOE Key Laboratory of Cell Activities and Stress Adaptations, School of Life Sciences, Lanzhou University, Lanzhou 730000, China; xiangy@lzu.edu.cn

**Keywords:** Calcium (Ca^2+^), actin, ABPs, channels, pollen tube, ROP, CDPK

## Abstract

In plant cells, calcium (Ca^2+^) serves as a versatile intracellular messenger, participating in several fundamental and important biological processes. Recent studies have shown that the actin cytoskeleton is not only an upstream regulator of Ca^2+^ signaling, but also a downstream regulator. Ca^2+^ has been shown to regulates actin dynamics and rearrangements via different mechanisms in plants, and on this basis, the upstream signaling encoded within the Ca^2+^ transient can be decoded. Moreover, actin dynamics have also been proposed to act as an upstream of Ca^2+^, adjust Ca^2+^ oscillations, and establish cytosolic Ca^2+^ ([Ca^2+^]_cyt_) gradients in plant cells. In the current review, we focus on the advances in uncovering the relationship between the actin cytoskeleton and calcium in plant cells and summarize our current understanding of this relationship.

## 1. Introduction

In plants, temporary and spatial changes in cellular Ca^2+^ concentrations play vital roles in growth, development, and signal transduction, such as tip growth of pollen tubes and root hairs, stomatal movement, salt or osmotic stress response, temperature and hormone response, as well as beneficial and pathogenic associations with microorganisms [1,2,3,4,5]. Changes in Ca^2+^ concentration often serve as triggers for calcium sensors or adapters, such as calcium-dependent protein kinases (CDPKs), calcineurin B-like protein (CBL) family, and Ca^2+^-dependent ABPs [1,2,6,7,8]. However, the underlying mechanisms of decoding Ca^2+^ signals and changes of cellular Ca^2+^ are largely unknown. Recent advances suggest that the actin cytoskeleton plays an important role in both the upstream and downstream of Ca^2+^ signaling.

The actin cytoskeleton—consisting of two forms of actin, globular actin (G-actin) and filamentous actin (F-actin)—is highly conserved in eukaryotic cells [9,10,11]. The two forms of actin are dynamically converted and regulated by a plethora of actin-binding proteins (ABPs) [9,10,11]. This dynamic conversion and regulation are essential for a variety of plant physiological processes [11,12,13,14,15]. Ca^2+^ can remodel the actin cytoskeleton by directly binding to ABPs to activate or inactivate their activity, or by indirectly regulating their activity via calcium-stimulated protein kinases, such as CDPKs [11,16,17,18,19]. In addition, the actin cytoskeleton can alter cellular Ca^2+^ homeostasis by regulating the influx and efflux of Ca^2+^ [20,21,22,23,24,25]. For example, the dynamics of actin have been proposed to act upstream of Ca^2+^, adjust Ca^2+^ oscillations, and establish free cytosolic Ca^2+^ ([Ca^2+^]_cyt_) gradients in plant cells [23,24]. This review mainly focuses on the relationship between the actin cytoskeleton and calcium in plant cells.

## 2. The Actin Cytoskeleton Adjusts Calcium Homeostasis

In plant cells, the resting [Ca^2+^]_cyt_ is maintained in the submicromolar range (about 100 nM) under normal conditions, whereas [Ca^2+^] is 1–10 mM in the cell wall and vacuole. Moreover, even the endoplasmic reticulum (ER) is expected to contain large amounts of Ca^2+^ [1,2,26]. These Ca^2+^ stockpiles can be used for elevating the [Ca^2+^]_cyt_ level under stress conditions or growth signaling [1,2,26]. Once a stress trigger or growth signal is received, the cytosolic concentration of calcium in plant cells can increase suddenly to the micromolar level, but Ca^2+^ is toxic to plants if high levels of the ion remain in the cytosol for a long period [1,2,26]. Therefore, plants use their apoplast or intracellular organelles, such as the vacuole and ER, to take up and store excess Ca^2+^ [1,2,26]. In normal conditions, [Ca^2+^]_cyt_ homeostasis plays a vital role in a myriad of physiological functions, and changes in [Ca^2+^]_cyt_ result from the influx and efflux of external and internal Ca^2+^ stores [1,2,26,27,28]. The influx of extracellular Ca^2+^ occurs mainly based on the plasma membrane (PM) calcium-permeable channels, and the efflux of Ca^2+^ is due to its release from intracellular Ca^2+^ stores, such as those in the vacuole, ER, mitochondria, and chloroplasts [1,2,29].

Intriguingly, many studies have revealed that the dynamics of actin cytoskeleton act as a signal transducer, contributing to the regulation of calcium-permeable channel activity [30,31,32]. For example, in plant pollen tubes, Wang et al. reported that the actin depolymerization reagents cytochalasin D (CD) and cytochalasin B (CB) significantly increased [Ca^2+^]_cyt_ levels, and that this increase in [Ca^2+^]_cyt_ was abolished by the calcium channel blocker La^3+^ or Gd^3+^ [20]. Moreover, the effects of actin depolymerization reagents on the channel were prevented by pretreatment with phalloidin, a stabilizer of actin filaments [20]. In *Vicia faba* guard cells, Zhang et al. found that disruption of actin dynamics activated stretch-activated (SA) Ca^2+^ channels, while stabilization of actin filaments blocked the activation of these SA channels under stretching or hypotonic treatment [33].

The reorganization and dynamics of the actin cytoskeleton serve as a link that also transfers external signals to induce calcium influx. For instance, in response to cold stimuli, disruption of actin filaments leads to Ca^2+^ influx, whereas stabilization of actin filaments blocks the cold-induced Ca^2+^ influx in plant cells [34,35,36]. Changes in [Ca^2+^]_cyt_ concentration in response to gravity are also widely accepted in plant biology [37,38]. Toyota et al. reported that the increase in [Ca^2+^]_cyt_ through mechanosensitive (MS) channels induced by changes in the gravity vector was attenuated by actin-specific reagents, implying that actin dynamics adjust calcium homeostasis by regulating the activity of MS Ca^2+^-permeable channels [39,40]. Regarding the underlying molecular mechanisms, plant annexins are considered to be as unconventional Ca^2+^-permeable channels and are involved in many developmental and stress-related processes [41,42,43,44]. Based on a few studies, annexins, such as AtANN5 and AnxGb6, can bind to F-actin and may function as scaffolding proteins for calcium and the actin cytoskeleton [45,46]. Therefore, actin dynamics may regulate the activity of Ca^2+^-permeable channels by influencing the annexin-actin interaction [43]. [Ca^2+^]_cyt_ changes may also derive from the release of the ion from the mitochondria and vacuole [23,24,47,48]. A study on *Arabidopsis* root hairs indicated that disruption of actin dynamics by latrunculin B (Lat B) and jasplakinolide (Jas) decreased the Ca^2+^ concentration in the mitochondria and induced an instantaneous elevation of [Ca^2+^]_cyt_, followed by a continuous decrease [23]. Moreover, Ca^2+^ concentration gradients exist in mitochondria from the tip region to the basal region of the root hair, and this Ca^2+^ gradient can be disrupted by actin-specific reagents, such as Lat B and Jas [23]. These findings suggest that a highly organized and dynamic actin cytoskeleton is vital for maintaining calcium homeostasis in root hairs.

Actin dynamics and Ca^2+^ play important roles in response to salt stress in plants, and the Arp2/3 complex, a nucleation factor of actin filaments that consists of seven subunits, was shown to integrate these two components [24]. Zhao et al. found that the disruption of actin dynamics by Lat B treatment increased [Ca^2+^]_cyt_ in response to salt stress, and plants lacking the subunit proteins in the Arp2/3 complex showed enhanced increases in [Ca^2+^]_cyt_ in response to salt stress, decreased mitochondria movement, and hypersensitivity to salt. Furthermore, ARP2/3 complex promotes actin assembly around mitochondria and drives mitochondrial movement [24]. Similar to the mechanism in plants, Boldogh et al. demonstrated that the mitochondrial motility in yeast is driven by actin polymerization and that this process requires the Arp2/3 complex [47]. These reports suggest that actin dynamics regulate mitochondria-dependent Ca^2+^ homeostasis in response to salt stress. In stomatal cells, although the actin-specific agents Jas and Lat B have no effect on resting tonoplast efflux, these two agents have opposite effects on the tonoplast efflux responding to exogenous abscisic acid (ABA). When treated with exogenous ABA, Jas reduced the ABA-induced transient stimulation of tonoplast efflux, while Lat B enhanced it [48], suggesting that actin dynamics adjust cellular calcium homeostasis by regulating calcium efflux from the vacuole.

Taken together, the reports above suggest that actin dynamics act as upstreams of calcium and adjust cellular calcium homeostasis by regulating the influx and efflux of Ca^2+^.

## 3. The Actin Cytoskeleton Acts a Potential Downstream of Calcium Signaling

Calcium signaling also regulates the dynamics of the actin cytoskeleton [12,18,19,49,50]. Pollen tube elongation depends on actin cytoskeleton remodeling, and actin dynamics are associated with the oscillation of the Ca^2+^ concentration gradients at the tip region of the pollen tube [49,50,51,52,53,54,55,56], suggesting that the Ca^2+^ gradient might precisely regulate actin dynamics to promote tube growth. In the tip-growing cell (e.g., protonema in the moss *Physcomitrella patens*) high [Ca^2+^]_cyt_ promotes actin filament disassembly, but low [Ca^2+^]_cyt_ promotes the assembly of a tip-localized actin spot. Moreover, abolishing the Ca^2+^ gradient leads to dramatic actin accumulation at the protonema tip. Together, these data indicate that the tip calcium gradient regulates actin accumulation to promote tip growth [57]. Calcium signaling is known to regulate actin dynamics through three main pathways: 1, calcium directly binds to ABPs to precisely remodel the actin cytoskeleton; 2, calcium-stimulated protein kinases, such as CDPKs, phosphorylate ABPs to adjust their activity and thus regulate actin dynamics; and 3, the Rho family of small GTPases (ROP GTPases) signaling pathway mediates actin cytoskeleton regulation via Ca^2+^.

### 3.1. Calcium Directly Binds to ABPs to Regulate Their Activity and Effect on Actin Dynamics

Actin dynamics are regulated spatially and temporally by different classes of ABPs, such as G-actin sequestration proteins, nucleating proteins, severing, and depolymerizing proteins, bundling and crosslinking proteins and end-capping proteins [9]. The activities of ABPs are regulated by several signaling factors, such as calcium (Ca^2+^), pH, phosphatidylinositol (4,5) bisphosphate (PIP2), and phosphatidic acid (PA) [2,11]. Among these signaling factors, Ca^2+^ is the most important second messenger for the regulation of plant development and stress signaling [2,11].

**Figure 1:**
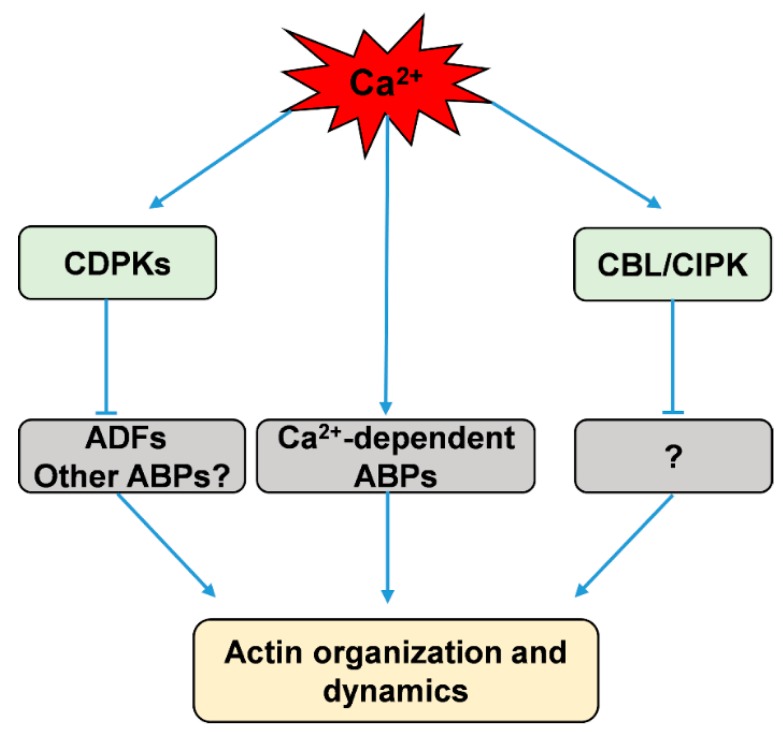
Simplified scheme showing calcium regulation of actin dynamics via direct and indirect pathways. CDPKs, calcium-dependent protein kinases; CBL/CIPK, calcineurin B-like protein and CBL-interacting protein kinases; ADFs, actin-depolymerizing factors; ABPs, actin-binding proteins.

From pollen hydration and germination to pollen tube tip growth, the process is accompanied by actin rearrangement regulated via numerous ABPs and calcium oscillations [14,15,58,59]. In dehydrated pollen grains, short actin fragments were the main form. In hydrated pollen grains, long, thin actin filaments appear. Upon germination, parallel actin cables encircle the shank of the newly growing tube and highly dynamic actin structures form in the tip [12,58]. During pollen tube tip growth, highly dynamic actin filaments are present in the apical dome; short actin filaments are arranged into a mesh ring or fringe-like F-actin structure in the subapical region; long, thick actin bundles are parallelly aligned in the shank region; and overall, a tip-focused calcium gradient is established in the pollen tube, in which maximum calcium concentrations can reach 1–3 μM in the tip [14,15]. Therefore, pollen tubes have been used as a model for the study of actin dynamics and calcium oscillations [14,15,59,60]. Over the past few years, based on this system, many studies have found that several Ca^2+^-dependent ABPs regulate actin dynamics in the tip growth of pollen tubes, including profilin, LIM domain-containing proteins (LILIM1 and PLIM2c), MICROTUBULE-ASSOCIATED PROTEIN18 (MAP18), MICROTUBULE-DESTABILIZING PROTEIN25 (MDP25), Rho-like GTPase of plants (ROP)-interactive and CRIB motif-containing protein1 (RIC1), and Villins (VLNs) [49,61,62,63,64,65,66,67]. In addition, in other physiological processes, a few ABPs, such as OsVLN2, VLN4 and fragmin-like protein, have also been characterized as regulating actin dynamics in a Ca^2+^-dependent manner [68,69,70]. These Ca^2+^-dependent/regulated ABPs are discussed below and summarized in Table 1 and Figure 1.

Among these Ca^2+^-dependent ABPs, the major group is the villin/gelsolin/fragmin superfamily, which is a class of multifunctional ABPs that remodel actin dynamics by nucleating, severing, depolymerizing, and bundling actin [86,87]. These ABPs typically possess two to six tandem gelsolin-like (G) homologous domains, which contain at least a conserved Ca^2+^-binding site, based on protein crystallography [86,88,89,90]. In plants, the first two villins to be characterized were 135ABP and 115ABP from lily (*Lilium*) pollen, which can bind and bundle actin filaments in a Ca^2+^-dependent manner [71,73,74]. There are five villin genes (*VLN1-VLN5*) in the *Arabidopsis* genome, and all of them bundle actin filaments in a Ca^2+^-insensitive manner [63,69,84,85,91]. In addition, with the exception of VILLIN1 (VLN1), these VLNs (VLN2-VLN5) exhibit nucleating, severing, and capping activities in a Ca^2+^-sensitive manner [63,69,84,85,91]. Moreover, there are two types of Ca^2+^ binding sites in the villin/gelsolin/fragmin superfamily, and as regards to gelsilin, Ca^2+^ binds type-1 sites at the interface of gelsolin is important for its interaction with actin, while Ca^2+^ binds type-2 sites buried within gelsolin activate this protein [92]. Khurana et al. found that all five VLNs in *Arabidopsis* have just one type-1 site in the G1 domain. In contrast to VLN1, which has only two type-2 sites in the G2/4 domains, VLN2-5 has one type-2 site in the G1 domain and another two to three type-2 sites in the G2/4/6 domains [84]. These results suggest that VLNs with a type-2 site in the G1 domain and a greater number of type-2 sites will be Ca^2+^-sensitive [84]. Furthermore, several studies demonstrated that a strong tip-focused calcium gradient is vital for VLN-mediated actin-filament severing to promote actin turnover, which may be correlated with actin dynamics and calcium gradients in pollen tubes [63,69,84,85,91]. In *Arabdopsis* root hairs, VLN4 caps and severs actin filaments in the presence of 5 μM Ca^2+^ but fails to do so in the presence of 0.5 μM Ca^2+^; these Ca^2+^ concentrations resemble those at the tip region and the shank region, respectively. Therefore, it may be that VLN4 needs to stabilize the actin cytoskeleton via its bundling activity in the shank region, while it promotes actin turnover by severing and capping actin filaments at the tip region [69]. To gain further insight, point mutations of calcium-binding site of VLN4 may be needed in the future. Moreover, Wu et al. also found that OsVLN2 exhibits conserved Ca^2+^-dependent actin filament severing, actin filament bundling, and actin filament capping activities. OsVLN2 promotes recycling of PIN2 and polar auxin transport by regulating actin dynamics to modulate morphogenesis of plant architecture [70].

In *Papaver rhoeas* (poppy) pollen, Huang et al. isolated and characterized an 80 kDa gelsolin-like protein, PrABP80, which possesses six tandem G domains and exhibits Ca^2+^-dependent actin-filament-severing and barbed-end-capping activities. This calcium-mediated actin filament depolymerization is vital for the self-incompatibility response in *P. rhoeas* pollen [80]. A fragmin-like protein with an apparent molecular mass of 42 kDa has also been identified in *Mimosa pudica*, which possesses three G domains, severs actin filaments, and enhances actin polymerization in a Ca^2+^-dependent manner [68]. From *Lilium davidii* pollen, the Ren group also identified a fragmin-like protein, LdABP41, which possesses three G domains, and the smallest member of this superfamily, ABP29, which possesses only two G domains; they found that these two proteins nucleate and sever actin filaments in a Ca^2+^-sensitive manner [12,75]. Recently, an ABP containing domains of myosin, villin, and GRAM (MdMVG) that directly binds and severs actin filaments in a Ca^2+^-dependent manner was identified in apples (*Malus domestica*). Moreover, MdMVG can physically interact with S-RNase, and this interaction inhibits the actin filament-severing activity of MdMVG in vitro. Therefore, S-RNase interacts with MdMVG to inhibit its actin filament-severing activity, while Ca^2+^ binds to MdMVG to enhance its severing activity and then regulates self-pollen tube growth during the early stage of self-pollination induction [93].

LIM is a family of proteins containing two LIM domains that essentially consist of two zinc fingers linked together by a short, two-amino-acid spacer and function as a module for protein–protein interaction [94]. Plant LIMs have been found to directly bind to actin filaments and bundle them into thick bundles [61,64,95,96,97]. To date, many LIMs have been identified in different species, including *Arabidopsis*, cotton (*Gossypium hirsutum*), lily (*Lilium longiflorum*), sunflower (*Helianthus annuus*), and tobacco (*Nicotiana tabacum*) [61,64,96,97,98,99,100], but only LILIM1 from lily and PLIM2c from *Arabidopsis* can bind to actin filaments and bundle them in response to Ca^2+^, and their activity is downregulated by high concentrations of Ca^2+^ [61,64]. One possible reason for this pattern is that LlLIM1 and PLIM2c are expressed in pollen tubes and regulate actin dynamics in response to calcium gradients in the pollen tube.

Two *Arabidopsis* PM-associated cation-binding proteins (PCaPs), namely, PCaP1 and PCaP2, which can bind to the PM and to calcium, have been proven to be microtubule (MT)-associated proteins, named MICROTUBULE-DESTABILIZING PROTEIN25 (MDP25) and MICROTUBULE-ASSOCIATED PROTEIN18 (MAP18), respectively [76,101,102,103,104]. Recently, these two MT-associated proteins were found to also regulate actin dynamics in pollen tubes [49,65]. Zhu et al. found that MAP18 exhibits Ca^2+^-dependent actin-filament-severing activity, and this activity is essential for determining the direction of pollen tube growth [65]. Moreover, MAP18 can modulate actin dynamics in tip-growing cells to regulate the direction of pollen tube growth and proper positioning of the nucleus in root hairs [65,105]. The second MT-associated protein, MDP25, also exhibits actin filament severing activity in the negative regulation of pollen tube growth. Furthermore, the calcium-binding site, formed by VEEKK residues, is responsible for Ca^2+^-dependent actin filament-severing activity [49,102]. Additionally, another MT-associated protein, RIC1, which belongs to the Rho-like GTPase of plants (ROP)-interactive, CRIB motif-containing protein family and which reorders MTs via promoting the MT-severing activity of katanin [106,107,108], was identified to regulate actin dynamics at the apical PM as well as the cytosol in pollen tubes [67]. Zhou et al. found that RIC1 could bind and cap the barbed ends of actin filaments and sever them in a Ca^2+^-dependent manner, and the distribution of RIC1 at the apical PM exhibits oscillation in concert with pollen tube growth. Moreover, high concentrations of Ca^2+^ enhance actin-filament-severing activity of RIC1 to regulate the abundance and oscillatory amplitude of fine actin filaments in pollen tubes [67]. Considering that the negative regulator of pollen tube elongation, MDP25, located at the apical PM, also exhibits actin filament severing activity [49], these studies suggest that Ca^2+^ might coordinate diverse actin filament severing proteins to regulate the proper organization, abundance, and dynamics of actin filaments for pollen tube tip growth [67].

Profilin as a key player in the early step of actin assembly is a G-actin binding protein with a molecular mass of 12–15 kDa capable of maintaining a pool of monomeric actin in cells [109,110,111,112,113]. The role of profilin is a condition-dependent dual function in actin polymerization and depolymerization [109,110,111,112,113]. Several studies have demonstrated that the binding of birch pollen profilin to muscle actin and the G-actin sequestering activity of maize profilin1/5 are regulated by calcium [81,82,83]. Myosin is a motor protein that uses energy to travel along actin filaments, and this actomyosin-based transport system is a key feature of cellular structure and dynamics, such as cell division and cytoplasmic streaming [114,115]. Yokota et al. first reported that Ca^2+^ could inhibit the travel of 170 kDa myosin along the actin filaments responsible for cytoplasmic streaming. Moreover, the light chain of myosin-CaM might be involved in Ca^2+^ regulation [78]. Annexin is another potential candidate that may link the actin cytoskeleton with calcium signaling in plants, since several vertebrate annexins have been proven to bind actin filaments in a Ca^2+^-dependent manner both in vitro and in vivo [116,117,118,119,120]. Similar to the annexins in animals, a few plant annexins have also been shown to bind actin filaments in a Ca^2+^-dependent manner, such as p34 and p35 from tomato, annexin from Mimosa, and AnxGb6 from cotton [45,77,79].

Collectively, these studies demonstrate that calcium can regulate actin dynamics by directly binding to ABPs to regulate their activity.

### 3.2. Calcium Indirectly Regulates the Actin Dynamics Via Calcium-Stimulated Protein Kinases, CDPKs

Posttranslational modifications are very important for the activity of some proteins [121,122]. Among various modifications, phosphorylation is widely involved in the activation and inactivation of ABPs in eukaryotes [123,124]. In plants, Ca^2+^ has been demonstrated to activate the CDPK family; the activated CDPKs subsequently transfer Ca^2+^ signals to downstream phosphorylation substrates to decode information related to Ca^2+^ oscillations and spikes [6,125,126]. The CDPK family was previously proven to colocalize with actin filaments, but CDPKs do not directly interact with actin [127,128], suggesting that CDPKs might interact with ABPs to localize to actin and that actin dynamics might be indirectly regulated by Ca^2+^ via activation/deactivation of CDPKs.

ADF/cofilin is a family of ABPs that can bind both G-actin and actin filaments and then depolymerize and sever actin filaments to promote rapid actin turnover [129,130,131,132]. Moreover, several two-dimensional electrophoresis studies revealed that both phosphorylated and unphosphorylated forms of ADFs exist in plant extracts from *Arabidopsis*, maize, tobacco, and moss [133,134,135,136]. The Hussey group found that maize actin-depolymerizing factor 3 (ZmADF3) could be phosphorylated at Ser-6 by CDPKs, and this phosphorylation inhibits an actin filament severing or depolymerizing activity of ZmADF3 [18,19]. However, which of the CDPKs phosphorylate these plant ADFs is not yet known [18,19]. Uno et al. used AtCPK4 and AtCPK11 as baits to isolate putative CDPK-interacting proteins by yeast two-hybrid (Y2H) screening, and their results showed an ADF among the CDPK-interacting proteins [137]. Recently, Dong et al. found that AtCDPK6 can phosphorylate AtADF1, predominantly at its Ser-6, and overexpression of AtCDPK6 can depress the activity of wild-type AtADF1 in severing/depolymerizing actin filaments, but not that of a mutant of AtADF1(S6A) in seedling root cells [138]. While ADF/cofilin is phosphorylated at Ser-3 in animal cells, the equivalent Ser-6 is conserved in plant ADFs [18,133], suggesting that ADFs could be phosphorylated at Ser-6 by calcium-stimulated protein kinases in plants. These results imply that calcium could activate CDPKs to phosphorylate ADFs and thus indirectly adjust actin dynamics (Figure 1).

Additionally, the CBL family, which serves as another Ca^2+^ sensor in plants, has been suggested to be involved in regulating the dynamics of the actin cytoskeleton [139]. CBLs perceive calcium signals to activate specific protein kinases, namely, CBL-interacting protein kinases (CIPKs); they then form CBL/CIPK complexes to relay the signals to downstream responses [8,140,141,142,143]. AtCBL4, also known as SOS3, which senses salt-elicited Ca^2+^ signals, interacts with and activates CIPK24/SOS2 to participate in salt-stress sensing and tolerance [144]. Ye et al. found that a loss of function in SOS3 disrupts the arrangement of actin filaments; actin assembly and arrangement in *sos3* are abnormal in response to salt stress, and external calcium or a low concentration of latrunculin A (Lat A) can partially rescue this phenomenon [139]. Collectively, these results suggest that the actin cytoskeleton is closely related to CBL/CIPK pathway that is possibly integrated by calcium signaling (Figure 1).

### 3.3. ROP GTPase Signaling Mediates Actin Cytoskeleton Regulation by Calcium

In plants, ROP GTPase, also called RAC-GTPase, plays a fundamental role in several important cellular processes, such as tip growth of pollen tubes and root hairs, regulation of the actin cytoskeleton, and hormone and stress response [107,145,146,147]. Similar to the Rho family proteins in fungi and mammalian cells, ROP GTPases regulate plant actin and MT cytoskeletal organization and dynamics [51,59,107,148,149,150]. ROP GTPases, serving as important molecular switches, mediate several signaling pathways in which calcium and the actin and MT cytoskeleton act downstream of the ROP GTPase signaling pathway [51,59,107,149].

**Figure 2:**
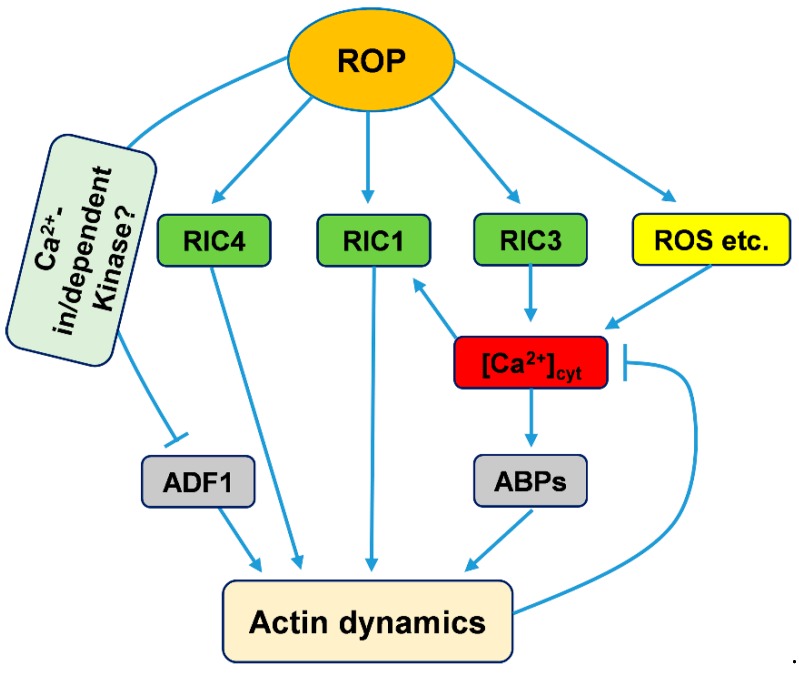
Working model to explain ROP signaling pathway mediation of actin cytoskeleton regulation by calcium in pollen tubes. ROP, ROP GTPases; RIC, ROP-interactive and CRIB motif-containing protein; ROS, Reactive oxygen species.

During pollen tube tip growth, ROP GTPase has been found to be involved in maintaining the Ca^2+^ gradient by interacting with reactive oxygen species (ROS), phosphoinositides, and pH gradient signaling in the tip regions of pollen tubes [59,150,151,152,153]. Furthermore, ROP GTPases can control Ca^2+^ gradients through their downstream plant-specific family of ROP effectors, RICs [52,146]. Two RICs, RIC3 and RIC4, adjust the tip Ca^2+^ gradients: RIC3 directly stimulates Ca^2+^ influx in the tube apex, while RIC4-mediated actin assembly might inhibit the accumulation of Ca^2+^ at the tip [56,146,154]. Gu et al. found that ROP GTPase could activate RIC3 and RIC4, which promote the formation of a tip Ca^2+^ gradient and the assembly of tip actin filaments, respectively. Then, elevated Ca^2+^ might induce actin disassembly through some ABPs, such as profilin or members of the villin/gelsolin/fragmin family [59,155]. Based on these results, there is a check-and-balance model encompassing ROP GTPase, RIC3 and RIC4 control of actin dynamics, the Ca^2+^ gradient and tip growth [56,155]. Together with the above study of RIC1, ROP GTPase regulates the organization and dynamics of actin mediated by Ca^2+^ gradients mainly via its downstream effectors, the RIC family (Figure 2).

Chen et al. found that NtADF1(S6A) with a nonphosphorylatable Ala substitution at the Ser-6 position shows high activity to counteract the inhibiting effect of NtRac1 overexpression on pollen tube tip growth, while the phosphomimic form of NtADF1(S6D) shows a reduced ability to counteract this inhibiting effect [135]. Moreover, overexpression of NtRac1 diminishes the actin-binding activity of NtADF1 but has no effect on the association of NtADF1(S6A) with actin filaments in pollen tubes [135]. Given that the phosphorylation of ADF/Cofilin inhibits its actin-filament-severing and depolymerizing activity, these reports suggest that ROP GTPase could regulate the organization and dynamics of actin through phosphorylation modification [135]. However, the mechanism underlying this phosphorylation remains unknown. In pollen tubes, the Hussey group showed that CDPKs could phosphorylate ZmADF3 to decorate its actin filament severing/depolymerizing activity, and that CDPKs can be activated by Ca^2+^ [18,19]. In addition, ROP GTPase promotes calcium accumulation in the tips of pollen tubes [59]. Overall, we speculate that ROP GTPase increases calcium levels to activate CDPKs, and then phosphorylates ADF to regulate its actin filament severing and depolymerizing activities.

## 4. Outlook

Overall, recent studies have shown that calcium can adjust the actin cytoskeleton by directly binding to ABPs and regulating their activity or by indirectly regulating their activity via calcium-stimulated protein kinases, such as CDPKs [17,18]. On the other hand, actin dynamics could maintain calcium homeostasis by regulating the activity of Ca^2+^-permeable channels [20]. Although tremendous progress in understanding the connection between the actin cytoskeleton and calcium has been made, many questions remain unanswered. The phosphorylation of ABPs by calcium-stimulated protein kinases is not well understood, and the calcium-stimulated protein kinases involved in phosphorylating ABPs and their phosphorylation substrates have yet to be identified. Moreover, it is unknown how the actin cytoskeleton regulates the activity of calcium-permeable channels, and one of the possible ways is that actin cytoskeleton adjusts their location on the membrane or the process of intracellular transport of calcium channels or receptors. In summary, future studies should focus on the signaling pathways through which calcium transduces environmental or developmental changes to the actin cytoskeleton and the mechanism(s) through which calcium homeostasis is regulated by the actin cytoskeleton.

## Figures and Tables

**Table 1:** Summary of calcium-dependent actin-binding proteins.

ABPs	Species	Tissues	Activities on Actin Organization	References
115ABP	*Lilium longiflorum*	Pollen tube	MF nucleation, capping, and bundling	[71,72]
135ABP	*Lilium longiflorum*	Pollen tube	MF nucleation, capping, and bundling	[73,74]
ABP29	*Lilium longiflorum*	Pollen tube	MF nucleation, capping, and severing	[12]
ABP41	*Lilium davidii*	Pollen tube	MF severing and capping	[62,75]
AnxGb6	*Gossypium barbadense*	Fiber	Actin binding	[45]
Fragmin-like 42-kD protein	*Mimosa pudica*	Petiole	MF severing	[68]
LILIM1	*Lilium longiflorum*	Pollen tube	MF bundling	[61]
MAP18	*Arabidopsis thaliana*	Pollen tube	MF severing	[65]
MdMVG	*Malus domestica*	Pollen tube	MF severing	[75]
MDP25	*Arabidopsis thaliana*	Pollen tube	MF severing	[49,76]
Mimosa annexin	*Mimosa pudica*	Pulvinus	MF binding	[77]
Myosin	*Lilium longiflorum*	None	MF binding	[78]
OsVLN2	*Oryza sativa*	Roots and Shoots	MF bundling, severing, and capping	[70]
P34/35	*Lycopersicon esculentum*	None	MF binding	[79]
PLIM2c	*Arabidopsis thaliana*	Pollen and Pollen tube	MF bundling	[64]
PrABP80	*Papaver rhoeas*	None	MF nucleation, capping and severing	[80]
Profilin	*Zea mays*	None	Sequester G-Actin	[81,82,83]
RIC1	*Arabidopsis thaliana*	Pollen tube	MF severing and capping	[67]
VLN2/5	*Arabidopsis thaliana*	Pollen tube	MF bundling, severing and capping	[63,66]
VLN2/3	*Arabidopsis thaliana*	Sclerenchyma	MF bundling, severing and capping	[84,85]
VLN4	*Arabidopsis thaliana*	Root hair	MF bundling, severing and capping	[69]

MF indicates actin filaments.

## Abbreviations

ABA	abscisic acid
ABPs	actin-binding proteins
ADF	actin-depolymerizing factor
CB	cytochalasin B
CBL	calcineurin B-like protein
CD	cytochalasin D
CDPKs	calcium-dependent protein kinases
CIPKs	CBL-interacting protein kinases
ER	endoplasmic reticulum
F-actin	filamentous actin
G-actin	globular actin
Jas	jasplakinolide
Lat A	latrunculin A
Lat B	latrunculin B
MAP18	MICROTUBULE-ASSOCIATED PROTEIN18
MDP25	MICROTUBULE-DESTABILIZING PROTEIN25
MS	mechanosensitive
MT	microtubule
PA	phosphatidic acid
PM	plasma membrane
PIP2	phosphatidylinositol (4,5) bisphosphate
ROP GTPases	The rho family of small GTPases
ROS	reactive oxygen species
SA	stretch-activated
VLN	VILLIN
Y2H	yeast two-hybrid
MDPI	Multidisciplinary Digital Publishing Institute
DOAJ	Directory of open access journals
TLA	Three letter acronym
LD	linear dichroism
